# Supplementary material for: Conformation of Polyethylene Glycol inside Confined Space: Simulation and Experimental Approaches
Source: Nanomaterials (Basel). 2021 Jan 19;11(1):244. doi: 10.3390/nano11010244 (PMC7832411; doi:10.3390/nano11010244)
Supplement: Supplementary file 1 [file nanomaterials-11-00244-s001.pdf]

Supplementary Materials:

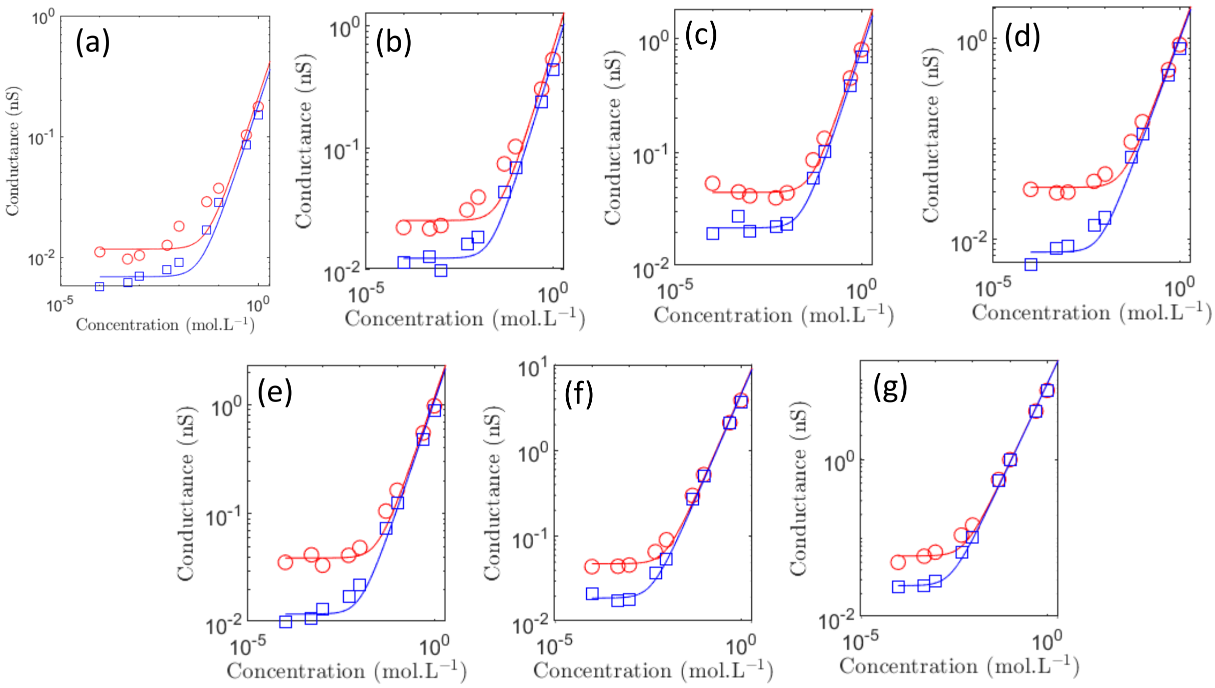

Figure SI-1: Conductance as a function of KCl concentration for cylindrical nanopore before (black square) and after (red, circle) with various diameters (a) 32 nm, (b) 40 nm, (c) 44 nm, (d) 41 nm, (e) 85 nm, (f) 117 nm

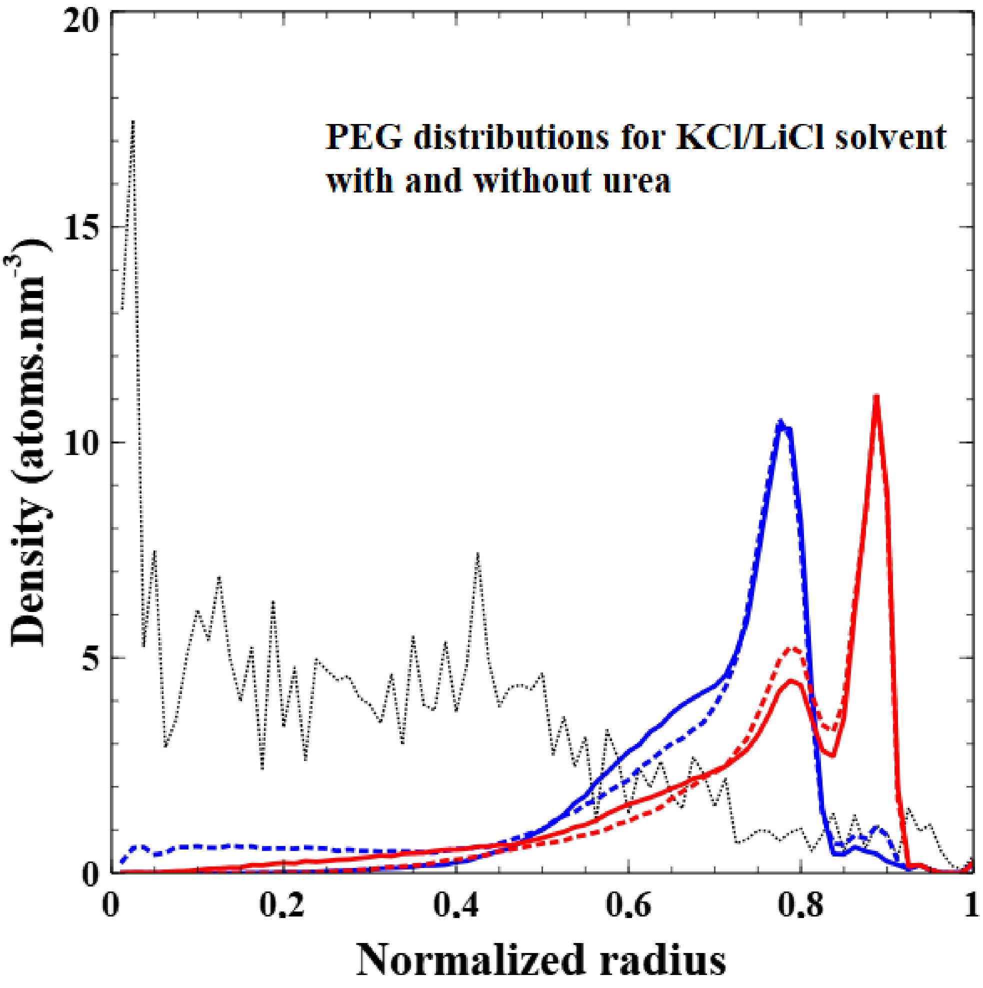

Figure SI-2: PEG distributions as a function of the salt used in the simulations. Full line for KCl and dashed lines for LiCl salt. In blue, simulations performed with urea and in red, simulations performed only with water.

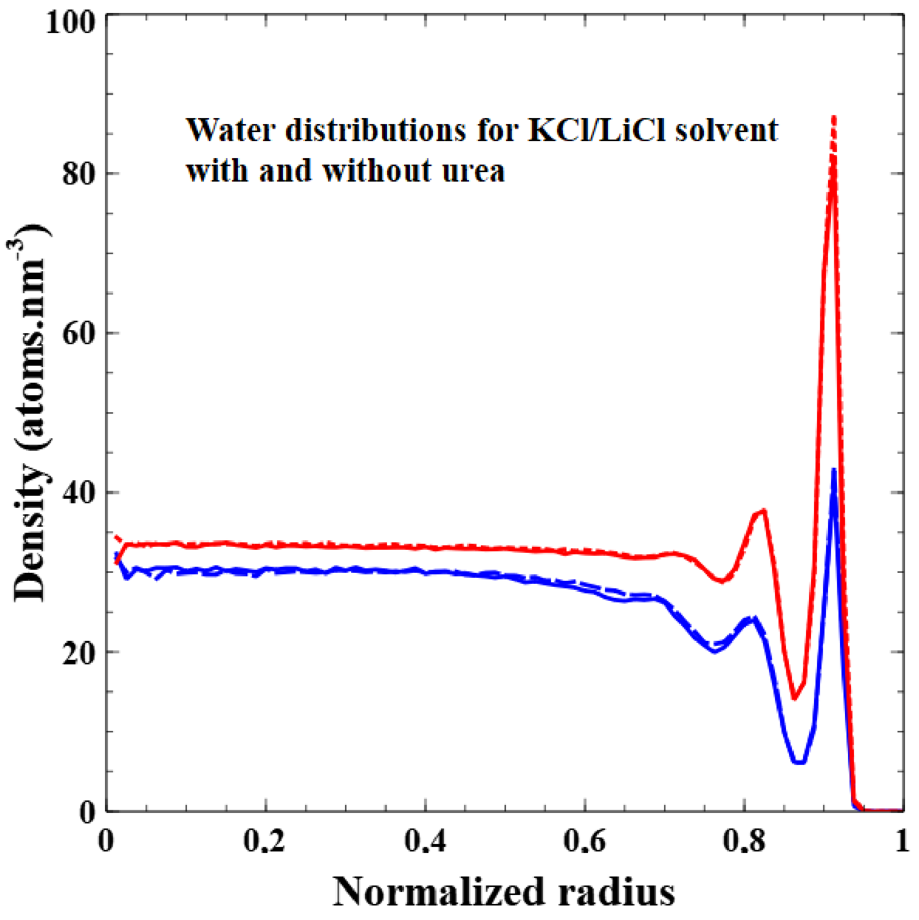

Figure SI-3: Water distributions as a function of the salt used in the simulations. Full line for KCl and dashed lines for LiCl salt. In blue, simulations performed with urea and in red, simulations performed only with water

Distribution

Radial distributions are computed using coordinates of each atom present inside the nanopore. It acknowledges the conicity of the pore, as each atom gets its radial coordinate normalized in accordance to the actual radius at their position along the nanopore. This normalized position is computed for 3 ns of simulated time and then averaged. Values are stored for every atom of each species -which are either K^+ or Li^+, Cl^-, urea, water or PEG- and are used to form a histogram. The number of atoms in every bin is then divided by the surface corresponding to the area between the 2 radii defining each bin, giving a density of atom by surface unit over the whole radius of the pore.
